# On the origin of metal species in the human brain: a perspective on key physicochemical properties

**DOI:** 10.1093/mtomcs/mfaf004

**Published:** 2025-02-08

**Authors:** Jake Brooks, James Everett, Peter J Sadler, Neil Telling, Joanna F Collingwood

**Affiliations:** School of Engineering, University of Warwick, Coventry, United Kingdom; School of Engineering, University of Warwick, Coventry, United Kingdom; School of Pharmacy and Bioengineering, Keele University, Staffordshire, United Kingdom; Department of Chemistry, University of Warwick, Coventry, United Kingdom; School of Pharmacy and Bioengineering, Keele University, Staffordshire, United Kingdom; School of Engineering, University of Warwick, Coventry, United Kingdom

## Abstract

Normal functioning of the human brain is dependent on adequate regulation of essential metal nutrients. However, it is also highly sensitive to metal-mediated toxicity, linked to various neurodegenerative disorders. Exposure to environmental metal sources (especially to particulate air pollution) can stimulate toxicity and neuropathologic effects, which is particularly evident in populations chronically exposed to high levels of air pollution. Identifying the sources of metal-rich deposits in the human brain is important in not only distinguishing the effects of environmentally acquired metals from endogenous metal dysregulation, but also for tracing pollutant sources which may be subject to exposure control. This perspective reviews evidence for key physicochemical properties (size/morphology, chemical composition, oxidation state, magnetic properties, and isotopic composition) concerning their capacity to distinguish sources of metals in the brain. The scope for combining analytical techniques to study properties in tandem is also discussed.

## Graphical Abstract

**Graphical Abstract:**
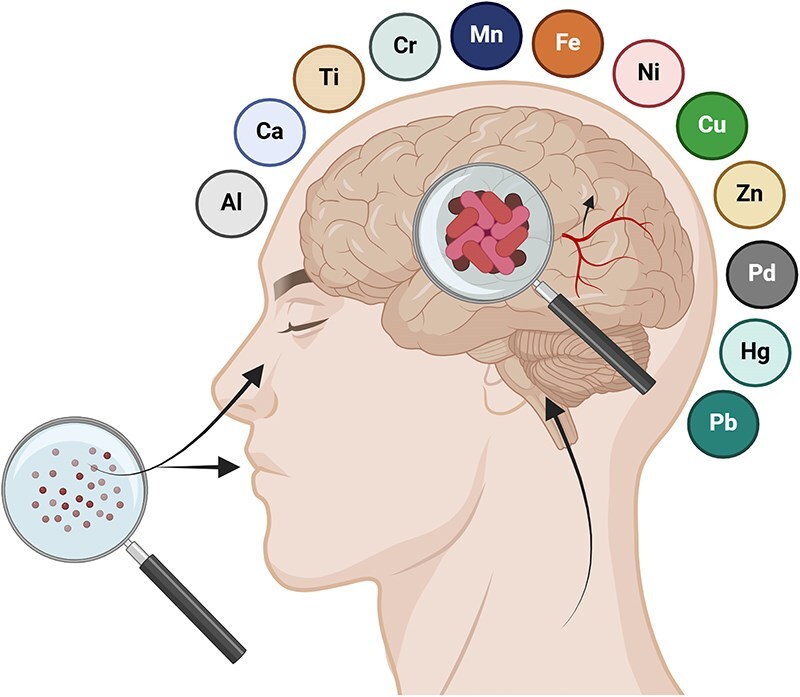

## Introduction

Of the elements which are essential to humans, many have known functions in the central nervous system (CNS), and numerous inorganic elements, including iron, copper, zinc, calcium, manganese, and selenium are transported to the brain as required for normal neuronal activity [1, 2]. The brain is regarded as a major metals repository where individual regions require a unique complement of metals for normal daily function [3].

Dysregulation of essential metals is associated with numerous neurodegenerative disorders, including Alzheimer’s disease, Parkinson’s disease, multiple system atrophy, amyotrophic lateral sclerosis, amongst others [4–8]. To date, Alzheimer’s disease has been a particular research focus in this field, with interactions between metals and the hallmark amyloid pathology frequently at the centre of investigations [9–14]. Metals can disrupt metabolic processes via numerous mechanisms, including redox imbalance, interaction with sulfhydryl groups in proteins, substituting and competing with essential metals for enzyme binding sites, receptors, and transport proteins [15, 16]. Furthermore, environmental or occupational exposure to xenobiotic metal sources (such as air pollution) has also been linked to the aetiology of neurological disorders [17–19]. It is thought that environmentally acquired particulates may act as catalysts for the formation of reactive oxygen species, as well as aberrant protein aggregation and fibril formation which form hallmark pathologies of neurodegenerative disorders [19]. This may even begin in early childhood [20, 21]. Although it is not yet entirely clear which components of particulate matter (PM) pose the greatest public health threat, animal models and studies of postmortem human tissue indicate that chronic exposure to metal-rich nanoparticles is likely to be of considerable concern [22–24].

### Metal transport into the brain

Upon absorption into the blood, metals are systemically distributed throughout the body, with the brain demanding a high proportion for normal physiological functions. The blood–brain barrier (BBB) and blood-cerebrospinal fluid barrier (BCB) in many respects isolate the CNS from the rest of the body—serving as a security system evolved to prevent passage of deleterious molecules from entering the CNS. Forming the BBB, a monolayer of specialized endothelial cells connected by tight junctions around the vascular lumen of the capillaries create a tight seal between the blood and surrounding neuronal tissues [15, 25]. Transporter proteins (similar to those found in the gastrointestinal tract) enable passage of metals across the BBB, and once inside the CNS, across cell membranes. For example, the divalent metal transporter DMT1 transports essential metals iron, copper, zinc, and manganese. Metal transporters lack high levels of specificity, an evolutionary feature which facilitates efficient transport of multiple cargos [15]. However, this lack of specificity may be exploited by non-essential, and often toxic metals, enabling molecules with similar physical and/or chemical (physicochemical) properties to hijack these distribution routes—termed ‘molecular mimicry’ [15, 17, 26]. For example, neurotoxic Cd^2+^ and Pb^2+^ may also be carried by DMT1. The BCB is formed by fenestrated blood vessels and networks of tight-junctioned epithelial cells at the choroid plexus, where CSF is synthesized [15, 25]. Many of the metals which enter through the BBB may also pass into the brain via the BCB [15].

The integrity of the BBB can be compromised by aging and disease. For example, BBB permeability increases with raised levels of systemic inflammation, and the effect may become more pronounced in the presence of pre-existing neuropathology (e.g. from Alzheimer’s or vascular dementia) [27]. This consequently may open the door to inadvertent metal uptake. Thought to be driven by blood-borne substances (e.g. metabolites, hormones, or cytokines), dynamic changes in BBB permeability can also occur in response to nutritional state, development, pregnancy, or psychological stress [28]. Cadmium neurotoxicity has been reported to occur during fetal development prior to complete BBB formation, or in connection with BBB dysfunction. Transport of cadmium across membranes is thought to be mediated by molecular mimicry, with cadmium binding to transporters and receptors for copper and zinc [17]. Elevated lead accumulation in the brain has been observed in children relative to adults, attributed to enhanced BBB permeability and lower bone storage capacity for lead. Incidentally, lead itself is also known to have an adverse effect on BBB permeability [15]. The extent to which BCB permeability and integrity contributes to these scenarios is less documented, and important to consider [25, 29, 30].

### Exogenous metals

Human exposure to environmental neurotoxic metals and metalloids is a significant global health concern, linked to the development of neurological disorders in both early life (e.g. autism) and in late life (e.g. Alzheimer’s and other forms of dementia) [31]. Significant human exposure to metals results from a range of anthropogenic sources, including mining, foundries, vehicles, and pesticides [32]. PM forms a class of air pollutant frequently associated with elevated risk of neurotoxicity and neurodegeneration [33, 34]. PM consists of an air-suspended mixture of particles of varying size and chemical composition, often rich in a variety of metals [35, 36] and further sub-classified based on maximum aerodynamic diameter e.g. PM10, PM2.5, and PM0.1 have maximum diameters of 10, 2.5, and 0.1 µm, respectively. Of increasing public health concern are metals that exist in nanoparticulate form, which may be inhaled if they form aerosols, and subsequently follow less guarded routes into the human brain. The smallest particulates (with diameters <100 nm) are interchangeably termed ultrafine particulates (UFPs), nanoparticles or PM0.1 [37]. Aluminium, calcium, chromium, iron, potassium, sodium, nickel, lead, titanium, vanadium, and zinc are reportedly the most abundant metals in UFPs, while less abundant elements such as cerium and uranium may also be present [37]. Combustion processes, of either natural or anthropogenic origin, are thought to be the principal source of UFPs [37], with risk of inhalant exposure elevated for certain occupations, particularly miners, welders, smiths and professions which incorporate metalwork [15, 38]. The concern surrounding metal-containing nanoparticles has, in recent years, been intensified by surging commercial interest in engineered metal-containing nanoparticles, which includes nanoparticles in metallic (zero-oxidation-state), metal oxide (e.g. magnetite), and metal alloy (e.g. Fe–Cr) forms. The unique properties of metal-containing nanoparticles are highly customisable and adaptable for a broad range of applications, including electronics, catalysts, and antimicrobials [39].

**Figure 1.**
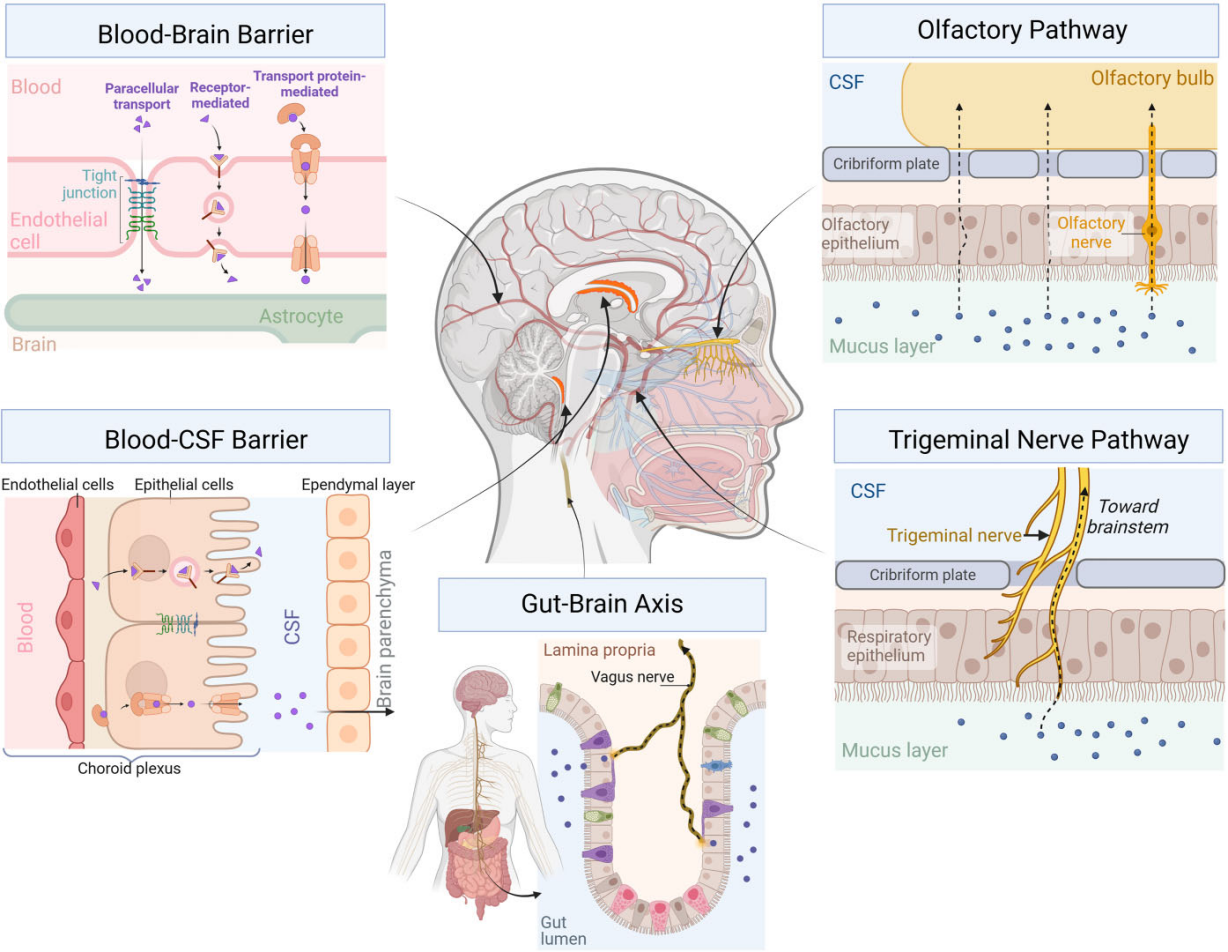
Entry portals for particulates to reach the brain. The blood–brain and blood–CSF barriers serve as entry points for particulates in systemic blood circulation. The olfactory and trigeminal nerves constitute neuronal pathways for inhaled particulates. The gut-brain axis (via the vagus nerve) provides a pathway for ingested particulates. Figure and associated graphical abstract created in BioRender. Brooks (2025) https://BioRender.com/h51x207 (28th February 2025, date last accessed).

Once inside the body, nanoparticles have propensity to translocate across cell barriers, as well as undergo neuronal transport, where retrograde and anterograde transport in axons and dendrites has been described [22]. The smallest particulates may therefore follow various routes into the brain; translocation and biokinetics are largely dependent on physicochemical properties, particularly particle size and surface chemistry [22]. The portals of entry to the brain are illustrated in Fig. 1, which have also been explored as a means for drug delivery to the CNS [40].

The interface with the external environment also leaves the brain vulnerable to unintentionally inhaled pollutants. Particles may penetrate into the gas exchange regions of the lung and subsequently pass into systemic circulation, from which it is possible to hijack transport mechanisms to cross the BBB via transcytosis [37, 41]. However, there is a more direct route for particulates to enter the CNS, via the olfactory/trigeminal pathway. Nanoparticles inhaled through the nose may undergo axon-mediated transport via the olfactory/trigeminal nerves into the brain, bypassing the BBB entirely [22, 42–44]. This has been demonstrated in numerous rodent studies, with one study evidencing around an eight-fold higher mass accumulation for nanoparticles entering via the olfactory pathway than the more indirect lung-bloodstream-BBB pathway [45]. An additional bypass for mediated transport of ingested metals is via the neuroenteric pathway (aka ‘gut-brain axis’), which has been demonstrated as a key portal to the brainstem via the vagus nerve [20, 46, 47].

### Scope

While the metabolism of essential metals is relatively well documented, there are still significant knowledge gaps concerning the entry and retention of exogenous particles into the CNS [41, 48]. Identification of exogenous particulates in the human body is a recognized analytical challenge [49], complicated further by evidence that metal-rich deposits of exogenous and endogenous origins can share similar physicochemical properties. Without clear distinction it is difficult to accurately assess the impact of external factors such as pollution exposure on the inorganic composition and function of the human brain. Moreover, the portals of entry into the CNS and specific characteristics of metal-rich deposits are likely to be significant in determining which body regions, cells, and organelles will be affected by inadvertent metal accumulation [20].

It is not the intention of this review to explore either the distinct physiological roles of individual metal elements, the mechanisms by which metals (essential or otherwise) may induce toxicity in the brain, or the myriad sources of environmental metals and their associated health risks. These topics have been thoroughly reviewed elsewhere [e.g. (15, 17, 31, 50, 51)]. The intention is instead to assess the utility of key physicochemical properties for determining the source of metal-rich deposits in the human brain i.e. whether they formed biogenically within the individual concerned or were inadvertently environmentally-acquired. This will facilitate better understanding of neurometallomics, as well as informing future policy development on limiting harmful levels of metal exposure.

## Characteristics of brain metal deposits

### Size and morphology

The propensity for metal-based particulates to evade regulatory biological defences increases with decreasing particle size; the highest particulate concentrations in the brain are expected to be achieved with the smallest particle sizes, while larger inhaled particles are more likely distributed elsewhere within the body (e.g. the respiratory tract) [22, 48, 49]. An upper size limit of 200 nm has been suggested for translation along the olfactory/trigeminal pathway into the human brain [42]. The same size threshold has also been suggested for penetrating gaps in the BBB [52], with studies targeting drug delivery across the BBB typically utilizing nanoparticles with diameters 50–100 nm [53]. Morphological analysis of candidate deposits requires probes enabling nanofocus spatial resolution, with high resolution electron microscopy-based approaches offering potential to reach atomic resolution imaging in combination with spectral information or elemental mapping [14, 20, 54].

In the context of exogenous airborne pollutants, this shifts the focus to PM in the smallest recognized size category, ultrafine (<100 nm diameter) and the finest PM2.5. Particles formed from condensation of gas precursors during combustion processes are approximately spherical, composed of organic and inorganic species. Initially <10 nm in diameter, such particles grow by coagulation and surface growth into larger particles with diameters of 100 nm or more [37]. Indeed, the characteristic morphology of inhaled magnetite/maghemite nanoparticles has been reported as rounded, with interlocking surface crystallites in the size range 10–100 nm [55]. In contrast, biogenically produced magnetic nanoparticles, frequently associated with biomineralization of magnetite by magnetotactic bacteria and linked with sensing of the Earth’s magnetic field, have been reported as euhedral particles with sharp facets, mostly in the size range of 10–70 nm [55, 56]. Both euhedral and rounded magnetite deposits were reported in frontal cortex tissue from Manchester- and Mexico City-based cohorts [57], with biogenic and exogenous sources attributed, respectively [56, 57].

A constraint on the use of only particle size and morphology to identify a structural fingerprint, is that environmentally acquired material may undergo agglomeration/de-agglomeration or partial dissolution either inside the body, or—depending on the method of analysis—during sample extraction. Consequent effects on particle sizes, concentrations and distributions may have a significant effect on the amount of metal-rich material which can be characterized, as well as the source attribution [48].

**Figure 2.**
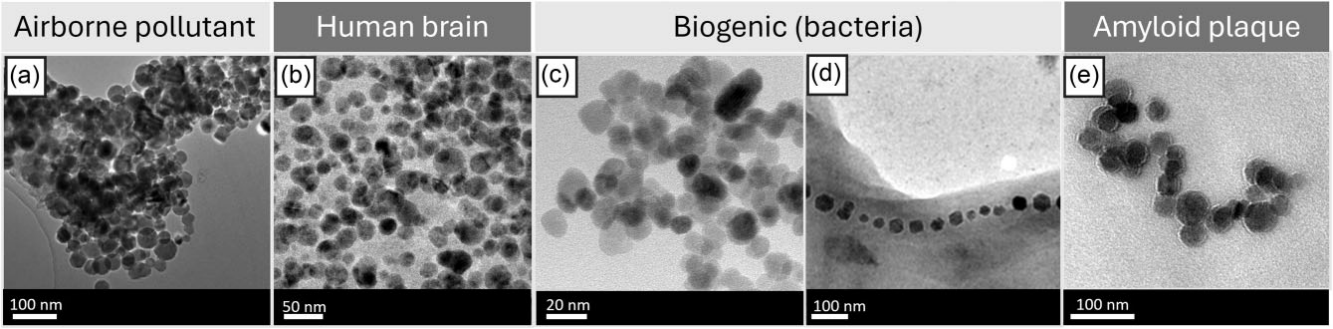
TEM images of iron oxide nanoparticles with different origins. (a) Iron oxide agglomerates collected from a roadside environment, Sanderson et al. 2016 [67]; (b) magnetite nanoparticles magnetically-extracted from human brain from Mexico City cases, Maher et al. 2016 [57]; (c) magnetite nanoparticles produced by by Geobacter sulfurreducens Byrne et al. 2011 [68], (d) chains of magnetite nanoparticles (magnetosomes) formed by Magnetospirillum gryphiswaldense, Staniland et al. 2007 [60] Copyright (2007) National Academy of Sciences, USA; and (e) Lorentz microscopy of magnetite nanoparticles within an amyloid plaque core isolated from Alzheimer's brain tissue, Plascencia-Villa et al. 2016 [14]. Panels a–e are reproduced with publisher permission.

Additionally, it is also possible for biogenic magnetite nanoparticles to exist both as small, rounded nanoparticles and as irregular shapes (see Fig. 2). Biogenic processes (such as those used by many bacteria) can mimic synthetic production as the process is fundamentally equivalent, i.e. particle nucleation followed by growth. Notably, the size and shape of biosynthesized nanoparticles can be altered by changing reaction conditions such as pH, temperature, concentration of precursor solution [39, 58]; e.g. gold nanoparticles biosynthesized using the bacteria *Rhodopseudomonas capsulata* were found to be rounded nanoparticles in the range of 10–20 nm at pH value of 7, and predominantly triangular nanoplates 50–400 nm in diameter at pH 4 [59]. Magnetotactic bacteria are different in this regard as the particle growth is constrained by intracellular vesicles, leading to more uniform and crystalline particles [60]. However, more than 10 000 microbial species have been estimated to occupy the human microbiome, leaving open the possibility that nanoparticles of various shapes and sizes could also be endogenously produced and translocated to the brain via systemic circulation or axon-mediated transport. Moreover, the long-held belief that the brain is a sterile environment is now under scrutiny, with some linking a brain microbiome to chronic neuroinflammation and neurodegeneration [61]. Even in multi-cellular organisms, biogenic magnetic nanoparticles can also occupy various shapes and sizes [62]. Magnetite nanoparticles of various sizes and morphologies have also been associated with amyloid plaques in Alzheimer’s brain [14, 63–65]. This includes rounded magnetite nanoparticles of diameter <10 nm, consistent with the ∼8 nm maximum diameter of ferritin cores [66]. The origins of such magnetite deposits have been related to aggregation of ferritin or the redox properties of amyloid beta (see also Section 3) [14]. Overall, these observations suggest that reliance on particles morphology in isolation may be insufficient to conclude a biogenic or exogenous origin (Fig. 2). As such, the study by Maher et al. investigated additional properties of magnetite nanoparticles reported in human brain [57] (see Section 6).

### Chemical composition

As descried earlier, the human brain requires a certain complement of metals to function. As such, the presence of elements with no recognized biochemical function may be considered a strong indicator of inadvertent uptake. While targeted studies of exogenous material in human brain tissue are relatively scarce, non-physiological metals in human brain have been evidenced. Quantitative bulk analysis techniques such as inductively coupled plasma mass spectrometry (ICP-MS), neutron activation analysis, atomic absorption spectroscopy (AAS), and X-ray fluorescence bulk analysis (XRF) have enabled sensitive detection of a wide range of elements in human brain [69–71], as reviewed by Ramos et al. [72].

However, bulk analysis methods cannot provide distributional information beyond the practical limit of tissue microdissection, and so chemical imaging is critical to contextualizing metal elements in the brain [73]. The combination of high-resolution electron microscopy with electron dispersive X-ray (EDX) spectroscopy (or EDX mapping) can be used to provide complementary information on the physical form and chemical properties of metal-rich deposits in post-mortem tissue. A catalogue of work documenting early-onset neurodegeneration in a Mexico City cohort has reported the presence of metal-rich nanoparticles in various brain regions (including olfactory bulb, frontal and temporal lobe, and cerebellum) from individuals chronically exposed to high levels of atmospheric particulate air pollution [20, 21, 57, 74, 75]. The substantia nigra, a region heavily affected by neurodegeneration in Parkinson’s disease pathogenesis, was shown to harbour rounded nanoparticle deposits rich in iron and aluminium and elongated particulates rich in titanium [20]. Metal-containing nanoparticles in substantia nigra were shown to associate with intracellular mitochondria and neuromelanin [20], which has well-recognized metal-binding capacity [76]. While iron is an essential brain metal, neither aluminium nor titanium have recognized functions in the brain. All three metals are, however, abundant in particulate air pollution. Ti-rich nanorods were also identified in neuroenteric neurons, suggesting entry via the gut-brain axis [20].

Both essential and toxic metals have also been identified among individual locus coeruleus (LC) neurons from multiple sclerosis cases, here using synchrotron XRF mapping. Varying concentrations of mercury, selenium, iron, copper, lead, bromine, and rubidium were observed among individual neurons [77]. Low concentrations of arsenic, cobalt, gold, chromium, manganese, nickel, platinum, and titanium were also observed in a few LC neurons in some cases [77]. As in the substantia nigra, neurons in the LC are also neuromelanin pigmented, possibly to protect against metal-mediated toxicity. Neuromelanin’s metal binding capacity may account for the selective metal accumulation in these cells and indicate history of metal exposure [78]. Retrograde axonal transport of toxic metals to the LC neuronal cell bodies from microvessels has also been suggested [79].

The wide-ranging use of metals in biomedical implants can under certain circumstances result in metal particulate or metal ion release (e.g. various forms of Cr, Co, and Ti from metal hip implants), into the surrounding milieu [80], which in some circumstances can lead to uptake into the brain. Although the most important source of exposure to organic mercury in humans is via fish consumption, it is the use of dental amalgams, composed of around 50% mercury by weight, that are recognized as the primary source of inorganic mercury exposure and resulting mercury vapour exposure affecting the CNS in the general population [81]. One study analysed mercury levels in 18 cadavers and observed elevated brain concentration of mercury in subjects with a greater number of occlusal amalgam surfaces (>12) compared with those with fewer occlusal amalgams [82].

The presence of non-essential metals, lead, cerium, platinum, and aluminium were reported in both Alzheimer’s and neurologically healthy control cases examined from the Manchester Brain Bank. All elderly cases were shown to contain concentrations of varying non-essential metals, with their presence putatively attributed to BBB compromise [55].

Detectable levels of aluminium have been observed in amyloid plaques isolated from Alzheimer’s brain tissue [65, 83]. The presence of aluminium more generally in the human brain has been reported, with typical levels described in aged brains donated to the Cognitive Function and Ageing Study [84]. More extreme examples include a case of cerebral congophilic angiopathy in an individual exposed to elevated aluminium levels in drinking water during the Camelford incident in 1988 [85]. It is understood that transferrin-receptor-mediated endocytosis provides a route for aluminium entry into the brain [86], and other metals such as gallium are also subject to transport via transferrin [87].

Particles identified as being of exogenous origin have also been reported in human CSF samples, including TiO_2_ (anatase), SiO_2_ and CaSnSiO_5_ (malayaite) nanoparticles [41]. Given the role of CSF in clearing waste products from the brain, it was assumed that exogenous material had originally entered the human brain via the BBB and was subsequently excreted as intact and agglomerated particles into the CSF. However, the authors of this work acknowledged that using CSF as a proxy indicator for metals in the brain is limited by the possibility that particles endocytosed and phagocytosed within the brain may be retained, and therefore not excreted into the CSF [41].

### Oxidation state

Chemically-reduced forms of iron and copper, as compared to the known forms utilized in normal iron and copper metabolism, have been associated with amyloid plaques isolated from humans with confirmed Alzheimer’s disease, including a fraction of mixed valence magnetite evident in Alzheimer’s tissue and amyloid plaques [64, 65] and trace levels of zero-oxidation-state metallic forms of both iron and copper [9, 63]. The initial rate of oxidation for iron nanoparticles is seen to increase exponentially with decreasing particle diameter [88]. It was thus deduced that unless surface oxidation or coatings had sufficiently stabilized such deposits originating from an exogenous source, that this chemical state observed in the amyloid plaque cores must otherwise be attributed to an endogenous pathway [9].

One possibility is that the observed nano-sized, chemically reduced deposits were formed via the well-documented ferriductase capacity of amyloid-beta for both iron in solution [89, 90], and mineralized forms of iron [91] such as the primary form of iron oxide material in the human brain, a ferrihydrite-like phase within the iron storage protein ferritin [66]. *In vitro* evidence has implicated amyloid beta interactions with ferritin in the formation of chemically reduced iron from ferritin’s inert ferric core, including Fe^2+^, mixed valence magnetite (Fe_3_O_4_), and zero oxidation state metallic iron (Fe^0^) phases [92]. Indeed, nanoparticulate ferritin-core-sized magnetite/maghemite crystalline material has been detected in amyloid plaques isolated from Alzheimer’s brain tissue [65]. Another possibility is biogenic formation by bacteria. Many organisms have the ability to bioreduce metal ions, coupled with enzyme oxidation [39, 93]. Such mechanisms have been harnessed by numerous species of bacteria as well as plants, fungi, viruses, and algae, and may be exploited commercially to biosynthesize zero-valent metallic nanoparticles with enhanced biocompatibility for biomedical applications, including drug and gene delivery, antimicrobials, and biomolecule detection [39, 58, 94]. Biosynthesis has been demonstrated for various metals, including palladium [95], gold [59], and copper [96]. Vodyanoy et al. also reported nanoparticles containing iron, copper, zinc, and other metals in human blood. Nanoparticles were reported as crystalline, metallic phases, with sources putatively attributed to dietary plants and gut bacteria [97].

### Magnetic properties

Magnetic nanoparticles are ubiquitous in the natural environment and can be produced by a range of anthropogenic activities [98]. The most abundant species, magnetite (Fe_3_O_4_) and its oxidized counterpart maghemite (γ-Fe_2_O_3_), are common constituents of atmospheric, urban PM pollution, identified in diesel exhaust, as brake-abrasion particles, and in the emissions from industrial combustion processes [56]. A recent study of particulate air pollution on the London Underground demonstrated predominantly 5–500 nm particles of maghemite, with superparamagnetic (<30 nm), single-domain (30–70 nm), and vortex/pseudo-single domain (70–700 nm) signals [99].

The abundance of iron-rich, magnetic nanoparticles in ambient PM has prompted use of magnetic characterization approaches to screen for elevated magnetic signal as a proxy measure for iron particulate exposure. One study used SQUID magnetometry to investigate ferrimagnetic material in various brain regions from neurologically healthy and Alzheimer’s cases (sourced from the Manchester Brain Bank), demonstrating the highest ferrimagnetic concentrations in the frontal lobe and reporting no significant difference between disease and control cases [55]. In the cerebellum, significantly higher ferrimagnetic concentrations (∼9×) were evidenced in a much younger Mexico City cohort compared to the Manchester cohort, consistent with prolonged exposure to much higher levels of particulate air pollution [20].

Use of magnetic properties to distinguish between endogenous and exogenous sources is limited by the fact that magnetite and its oxidized component maghemite are also naturally occurring iron oxide minerals. Crystals of these minerals have been reported in a wide range of animal species, with the suggestion that they could be involved in navigation via magnetic field detection [100]. The first report of magnetite and maghemite nanoparticles isolated from the human brain was by Kirschvink and co-workers [54]. Most larger particles were ∼200 nm, arguably consistent with the threshold for entry via the olfactory route, but magnetite and maghemite particles up to 600 nm in diameter were also observed, and the nanoparticles investigated in this original study exhibited crystallographic features, chemical composition, surface texture, prismatic particle shape, and associated magnetic properties (including [111] crystal alignment for the maximum saturation magnetization) all supporting the idea that these particles formed biogenically [54]. Another study observed increasing brain magnetite accumulation with age in males only, postulating that increased retention of dietary iron with age in the male population may cause increased magnetite biomineralization, possibly through iron overloading in ferritin or disruption to ferritin function [101]. In contrast, elevated concentrations of magnetite have been observed in Alzheimer’s brain relative to age-matched controls, where the highest concentrations were observed in female subjects with Alzheimer’s [102]. Ferritin malfunction has been implicated in the biosynthesis of magnetite in human brain by several studies [65, 103]. Others have postulated that precipitation of magnetite nanoparticles in the brain plays a key role in long-term memory [104].

Bulk magnetic measurements (such as SQUID magnetometry) provide no direct chemical or structural information, and the accuracy and precision for obtaining quantification have been questioned [105]. Further, magnetic separation lacks specificity for selectively extracting target magnetic phases (such as magnetite) from complex matrices, since other iron phases present in tissue (e.g. ferric salts) are also magnetisable [105].

### Isotope composition

Isotope tracking has long been used to monitor the uptake and metabolism of radionuclide-labelled metals in animal studies [106]. However, advancement of relevant analytical tools, particularly the introduction of multi-collector ICP-MS, has enabled smaller changes to be quantified in stable isotope compositions at ever decreasing concentration [107]. Such techniques can now be used to monitor intrinsic changes in stable isotope composition of various biometals, altered by differences in the reaction efficiency of their isotopes, through various metabolic processes. Thus, isotope fractionation can be used to track metabolism of essential metals such as iron, copper, zinc, and calcium and has thus been investigated as a potential biomarker for disease [108]; e.g. changes in the isotopic compositions of zinc and copper have been reported in Alzheimer’s brains, attributed to disrupted chemical bonding environment caused by hallmark amyloid plaque and tau fibril formation [109].

Single or multi-metal isotope fractionation has been exploited in the environmental sciences to identify isotopic signatures of different pollution sources [98, 110–112]. For example, stable iron isotopic composition was used to identify the source of magnetic PM in Beijing sandstorms as a mix of natural and anthropogenic emissions [110]. Negative isotope signatures characteristic of smelting and combustion gas emissions were used to indicate a primary source of atmospheric air pollution in Barcelona as being the metallurgical industry [111]. However, while widely applied to contamination tracing in air samples, soils, sediment, and application to source apportionment in biological samples is relatively scarce [113].

Several research teams have attempted to identify an isotopic signature for exogenous metals in biofluids. Cikomola et al. measured the isotopic composition of iron in blood serum collected from a sub-Saharan African population exposed to high levels of atmospheric iron concentration. Lower mean ^56^Fe concentrations (relative to European populations) were putatively attributed to enhanced uptake of iron from the surrounding environment [114]. Anthropogenic sources of copper, such as vehicle emissions, road dust, smelting-derived particulates, are generally enriched in ^65^Cu relative to geological sources [115]. This has been postulated as a possible explanation for elevated ^65^Cu in European and Japanese blood relative to that of the Yakut in the circumpolar Vilyuysk region of Russia [107, 116].

Various physiological and lifestyle factors constrain the use of isotope fractionation as a fingerprinting tool for metal origin. Influencing effects on isotopic composition were identified for genetic variations, diet, and obesity amongst others [107]. Such factors may be difficult to control for in studies of the human brain, as these are largely reliant on provision of postmortem tissue representing a single timepoint, and with limited access to supporting physiological and lifestyle information for each donor. Notably, the many changes in oxidation state required for normal absorption and storage of redox metals like iron and copper result in extensive isotopic fractionation as a consequence of normal metabolism [117]. Zinc is not a redox sensitive element and fractionates significantly without changes in oxidation state [117]. Indeed, Jaouen et al. demonstrated the use of zinc isotope ratios in blood samples to track dietary habits human populations [118]. Application to the study of metal origins in human brain has yet to be fully realized but the development of analytical tools such as single-particle ICP-MS (designed for the characterization of nanoparticles) coupled with multi-element and multi-isotope detection offers clear potential [119]. Indeed, the potential for stable isotopic analysis to be used as a source tracing tool for internalized PM has already been recognized [49], while wider study of elemental isotopic signatures in populations exposed to anthropogenic metal source has also been suggested [107].

### Correlative approaches

The analytical determination of each material property in isolation has limited capacity for distinguishing the origin of metal deposits in the brain, but probing multiple physicochemical properties in tandem has the potential to reveal greater insights. For example, combined application of electron microscopy techniques (High-resolution TEM, electron energy loss spectroscopy (EELS), and EDX) and SQUID magnetometry was used to attribute the presence of magnetite nanoparticles in human brain tissue to combustion and friction-derived sources based on their small size, rounded morphology, surface crystallites and colocalization with non-physiological metals [57]. This matches structural and elemental fingerprints for environmental magnetite pollutant particles identified using correlative scanning transmission electron microscopy, EDX, X-ray photoelectron spectroscopy, and electron diffraction. Indeed, pollutant magnetite nanoparticles showed fused interlocking surface crystallites with varying lattice orientations (characteristic of high-temperature formation and crystallization upon rapid cooling and/or oxidation) and also contained a wide range of additional trace metal elements, including Mg, Cr, Mn, Co, Ni, Cu, Zn, and Pd [105]. Co-localization of palladium with magnetite may indicate vehicular-derived source, given the ubiquitous use of Pd in catalysts (which are subject to wear during vehicle operation) and extremely low natural background concentration of Pd [105]. While palladium was not observed for magnetic nanoparticles identified in brain tissue, platinum was observed for some deposits, which may also be linked to use in catalytic converters [57]. Other metals associated with catalyst wear, including rare earths lanthanum, cerium, and praseodymium, have also been reported in brain tissue from the Mexico-city cohort [74]. Exact sources of the observed magnetic nanoparticles in the Mexico City cases could not be traced, although probing stable metal isotope signatures may help to identify a more precise source [98].

Correlative approaches have been employed to positively identify exogenous materials in other human-derived samples from compartments external to the CNS. For example, various properties including size (via electron microscopy), crystal structure (via X-ray diffraction), chemical composition (via EDX), and isotopic composition (via ICP-MS) were used to identify a chemical fingerprint for metal-rich combustion-derived particulates in human serum and pleural effusion samples [48]. In a different study, particulate TiO_2_ and SiO_2_ were observed in liver, spleen, kidney, and intestinal tissues using correlative spICP-HRMS to investigate element concentrations and SEM-EDX to measure particle size range/morphology. TiO_2_ and SiO_2_ particulates were assumed to originate from oral exposure, including food, toothpaste, and medicines as suggested sources [120].

Correlative workflows have also been applied to study metals in human brain tissue samples, although not necessarily with the intention of investigating the sources of each metal element. For example, analytical electron microscopy has been correlated with NanoSIMS to reveal the quantitative elemental composition and isotopic distribution of metals (including iron, copper, and calcium) in nigral neurons [121]. It is worth noting that where complementary analytical techniques are applied sequentially, the workflow must ensure initial analysis does not preclude subsequent measurements.

The advancement of non-destructive techniques towards ever increasing chemical sensitivity, spatial resolution, and specificity is transformative in enabling correlative analysis, not only facilitating better quality data but also permitting greater flexibility in how techniques can be combined. In particular, multi-modal chemical imaging at synchrotron facilities enables non-destructive analysis of various physicochemical properties at nanoscale spatial resolutions, while also meeting the sensitivity and specificity demands presented by trace element analysis in human brain tissue [73, 122]. Applications have frequently focussed on brain metal dysregulation linked to neurodegenerative disease. For example, X-ray magnetic circular dichroism has been combined with scanning transmission X-ray microscopy for site-specific magnetic and oxidation state characterization in nanoscale iron deposits associated with amyloid plaques isolated from Alzheimer’s brain, enabling iron species to be determined with precision [9, 63, 123]. Other studies of metal elements in amyloid plaques have combined synchrotron XRF with fourier-transform infrared spectroscopy (FTIR) and X-ray absorption near-edge spectroscopy to correlate plaque aggregation state with iron concentration and speciation [124], and with FTIR and Raman spectroscopy to study relative distributions of organic and inorganic plaque components [13]. Synchrotron XRF has also been combined with X-ray ptychography to characterize elemental fingerprints in pathological regions in Parkinson’s disease brain tissue [125], with fluorescence light microscopy under cryogenic conditions to correlate metal and protein distributions in developing neurons [122]. Various synchrotron beamlines also permit direct correlation with spatially resolved X-ray diffraction, which has been applied to study brain metal distributions in the context of pathological crystalline structures [126, 127] and has potential to be applied for refining the chemical speciation of environmental deposits [128].

### Analytical challenges

Locating nanoscale metal-rich deposits in vast areas of tissue remains a key analytical challenge. The development of lab-based tools for non-destructive screening of samples at microfocus resolution (e.g. using benchtop XRF) can aid identification of metal hotspots for subsequent interrogation using a nanofocus approach, which provides new opportunities [129, 130]. This is especially applicable to non-essential metals (e.g. lead, titanium), deposits of which would likely be identifiable (even if the pixel size is much larger than the deposit size) against their minimal background levels in tissue. Additionally, the emergence of new image analysis tools which utilize machine learning for automated detection and classification of particles based on morphology may form a key component of this correlative toolbox, helping to improve speed and accuracy of detection [131, 132].

It is also imperative that preparation approaches are used which limit the potential sources of sample contamination. This typically involves use of non-metallic tools in the cutting and sectioning of tissue samples and use of ultra-pure reagents. However, it is often impractical for preparation and analysis of tissue samples to take place under clean room conditions, particularly when incorporating multi-modal approaches and specialist analytical techniques such as those at synchrotron facilities. Site-specific analysis of tissue sections (as opposed to bulk analysis via acid digestion, e.g.) alleviates some vulnerability to sample contamination, since (i) the surface of the sample block can be removed during tissue sectioning and/or selectively excluded during measurement, to omit surface contamination that might have been introduced during initial tissue handling and (ii) the higher resolution microscopy approaches have limited depth of field, such that contaminant material deposited on the surface of a tissue section is in a different focal plane to the tissue ultrastructure. However, accurate visualization of the tissue during measurement is necessary to benefit from these possibilities. Knowledge of the sources of signal in the beam path is critical to X-ray microscopy, so that the sample can be analysed against background contributions. Depth-dependent variations may also require consideration, particularly where there are dense inclusions in a sample. For the primary organic components of biological tissue, typically sectioned at sub-mm thickness, a hard X-ray beam can be assumed to fully penetrate multiple layers [e.g. at 12 KeV; X-rays have penetration depth of >3 mm in biological tissue (73)].

It should also be noted that appropriate management of beam dosage is required for synchrotron techniques to be applied non-destructively, since X-ray induced sample alterations are often not observable to the naked eye. Chemical changes induced by irradiation of the sample using high intensity X-ray beams can include chemical reduction/oxidation, bond-rearrangements, or changes in the coordination geometry [133]. Minimizing X-ray dose is possible by optimizing scanning parameters on standards of known chemical state, while repeating sample scans to check for beam-induced changes can help to mitigate against such measurement artefacts [9, 134]. Element-specific dose limits have also been investigated for XRF use [135].

While the potential for application of correlative workflows to determine the origins and impact of metal deposits in human brain tissue is yet to be fully realized, the ongoing development and application of potentially non-destructive techniques such as those available at synchrotron facilities will accelerate the rate at which we can address these analytical challenges.

## Summary

Distinguishing between endogenous and exogenous metal sources in such a heterogenous and complex environment as the human brain is non-trivial; physicochemical profiles occasionally used to identify exogenous species can also be replicated biogenically, e.g. biogenic mineralization of magnetic iron phases and biogenic production of rounded metal-containing nanoparticles. Better understanding of the biological pathways by which environmental metals can enter and are handled within the brain will help to refine the regions of interest, not only to specific brain regions, but possibly to specific sub-layers or cell types. This will contribute to the nanoscale analysis required for multi-modal particle characterization. Probing multiple properties in tandem creates excellent opportunities for more precise and accurate identification of metal sources. With development in analytical tools (e.g. multi-element single particle ICP-MS) it may now be possible to more widely incorporate source apportionment techniques (e.g. isotope fractionation) applied in the environmental sciences to tracing the sources of metals in postmortem brain tissue. Additionally, development of non-destructive techniques such as synchrotron X-ray spectromicroscopy will benefit correlative workflows which permit consecutive analyses of the same sample, although care must be taken to ensure that sample handling and measurement does not incur changes in metal speciation. Advances in non-destructive screening and image analysis tools which incorporate machine learning may enhance opportunities for increasing measurement throughput. Tracing the sources of metals in the brain evidently remains an open frontier for study; however, further investigation in this area will enhance understanding of metal metabolism in the human brain and provide a scientific basis for environmental protection and metal pollution control.

## Data Availability

No new data were generated or analysed in support of this research.
